# Surgical ‘damage control’ treatment of a large retroperitoneal liposarcoma encasing a horseshoe kidney

**DOI:** 10.3332/ecancer.2008.77

**Published:** 2008-07-07

**Authors:** B Andreoni, A Chiappa, U Pace, E Bertani, F Verweij, F Orsi, G Petralia, M Tullii, M Venturino, G Pelosi

**Affiliations:** 1 Department of General Surgery, European Institute of Oncology, Milan, Italy; 2 Department of Urology, European Institute of Oncology, Milan, Italy; 3 Department of Radiology, European Institute of Oncology, Milan, Italy; 4 Department of Anaesthesiology, European Institute of Oncology, Milan, Italy; 5 Department of Pathology, European Institute of Oncology, Milan, Italy

## Abstract

Damage control is a surgical strategy for severely compromised trauma patients based on speed control of life-threatening injuries that aims to rapidly resuscitate patients in an intensive care unit (ICU). We report on the use of such therapeutic strategy in a patient affected by a retroperitoneal sarcoma concomitant to a horseshoe kidney, a relatively rare anatomical malformation.

## Introduction

Retroperitoneal soft tissue sarcomas (RPS) are rare mesenchymal tumours, their incidence being about 10% of all soft tissue sarcomas, which together constitute less than 1% of all malignant neoplasms [1]. Surgery is the principal mode of therapy and offers the most favourable prognosis after complete resection [2–4]. Complete resection is, however, often problematic to perform because of the large size of the tumour at the time of diagnosis, the difficult, deep-seated central location and common infiltration to adjacent organs.

We report a single, exceptional case of a patient affected by retroperitoneal liposarcoma, observed, diagnosed and treated in our department with a combined strategy of extreme surgery and damage control.

## Clinical case

A 67-year-old male was hospitalized on 26 September 2007 because of a large, paucisymptomatic retroperitoneal tumour. The patient had been presenting progressive abdominal swelling, weight loss and asthenia for three months.

**Figure 1:**
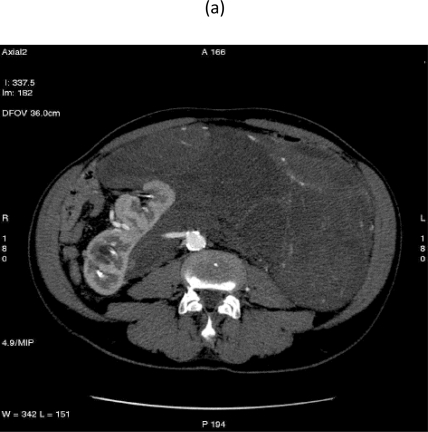

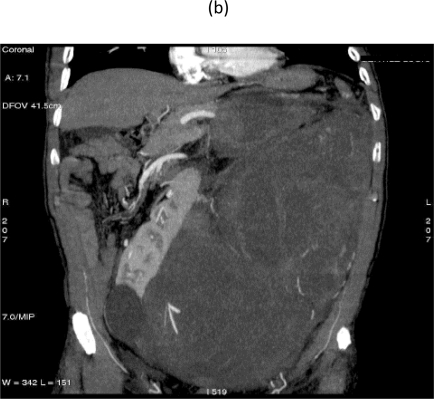
Retroperitoneal liposarcoma, as visualized in contrast-enhanced multidetector computed tomography (MDCT) of abdomen and pelvis. Multiplanar reformations (MPR) show the whole tumour extension, measuring 25 × 13 cm in the transverse plane (a) and 36 cm in the coronal plane (b).

**Figure 2:**
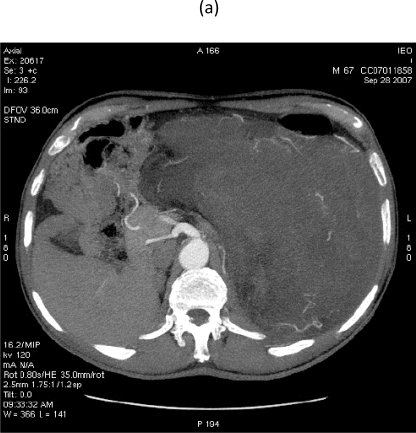

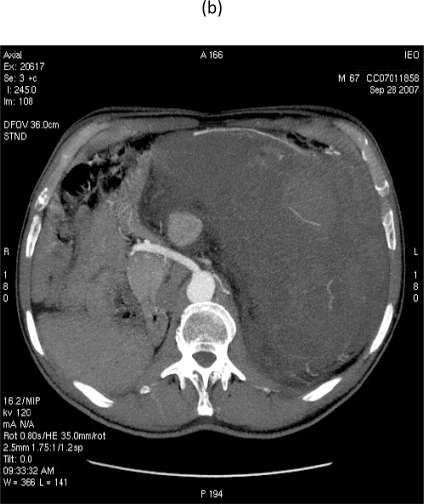

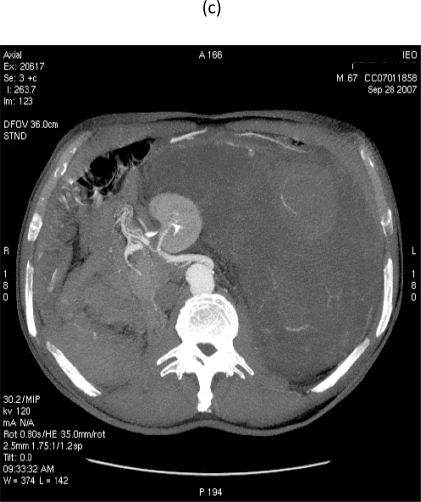

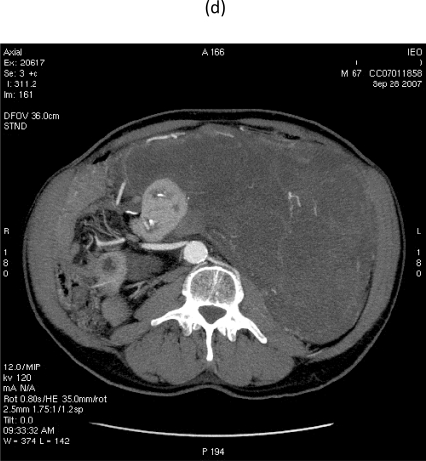

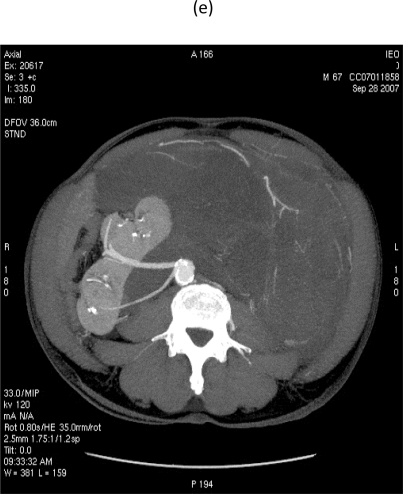

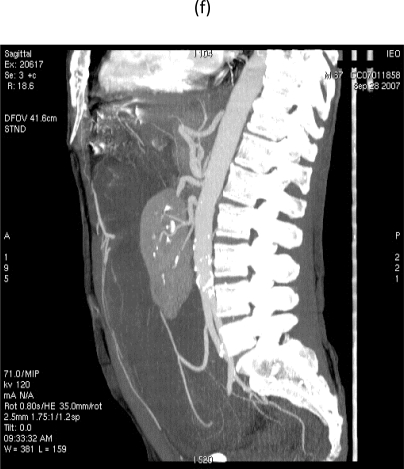

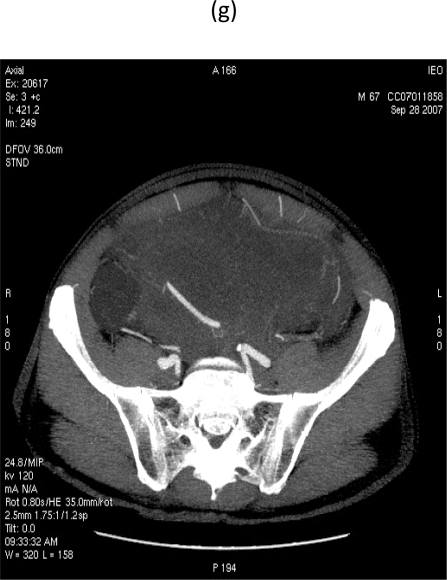
Images of MDCT angiography in the early-arterial phase show the origin of visceral arteries from abdominal aorta and common iliac arteries. From cranio- to caudal transverse planes, the origin of the following arteries is visualized: (a) celiac trunk, (b) superior mesenteric artery, (c) left renal artery, (d) inferior mesenteric artery, (e) double right renal arteries originating from aorta (f) supernumerary artery for the lower pole of the right kidney, originating from the right common iliac artery, (g) supernumerary artery for the lower pole of the left kidney, originates from the left common iliac artery left inferior polar renal artery and passes through the neoplastic mass.

**Figure 3:**
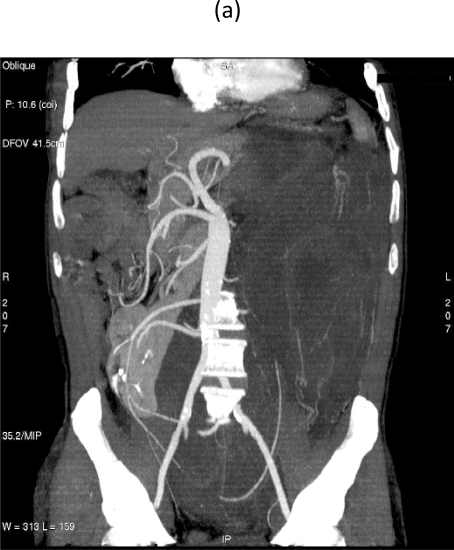

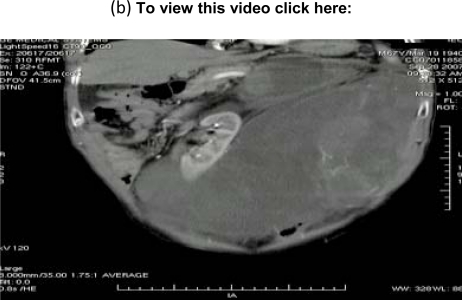

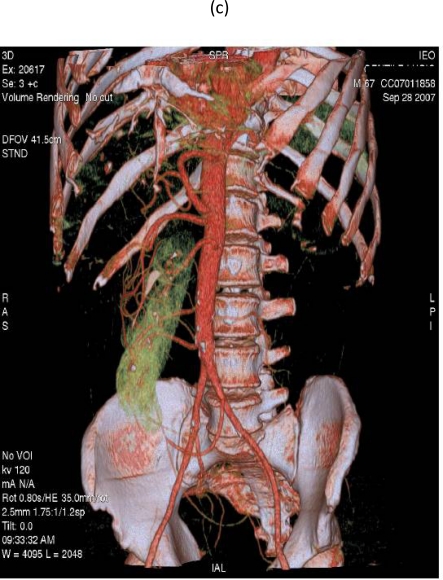
Maximum intensity projections (MIP) images (a–b) and volume rendering images (VR) (c) provide comprehensive evaluation of vascular anatomy. http://www.ecancermedicalscience.com/view-article.asp?doi=10.3332/ecancer.2008.77

**Figure 4:**
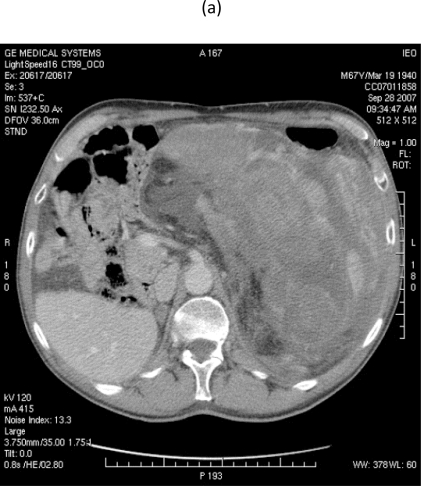

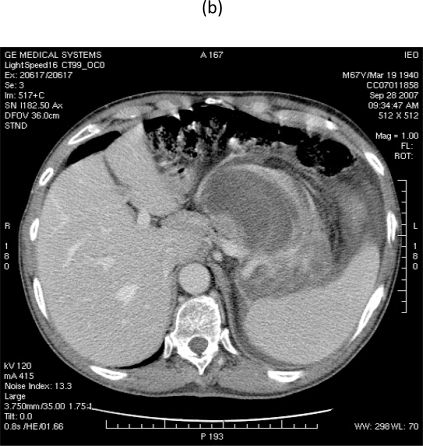

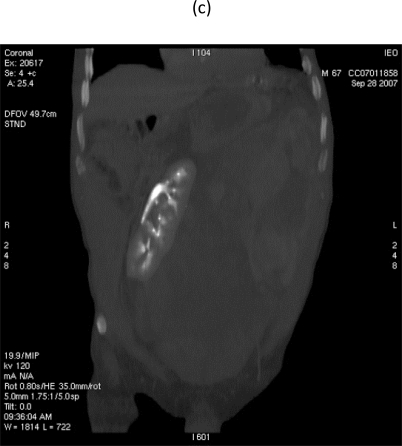
MDCT images show (a) dislocation of small bowel and colon in the absence of obvious infiltration and (b) dislocation and infiltration of the spleen; infiltration of horse shoe kidney is observed, in the absence of ureteronephrosis (c).

**Figure 5:**
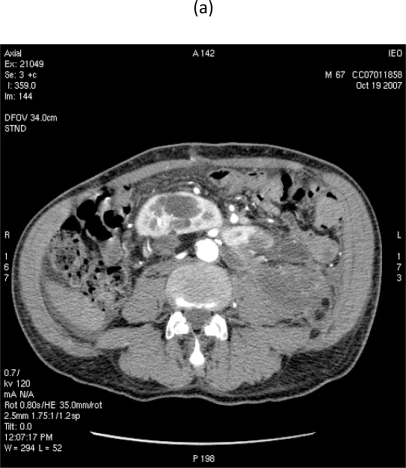

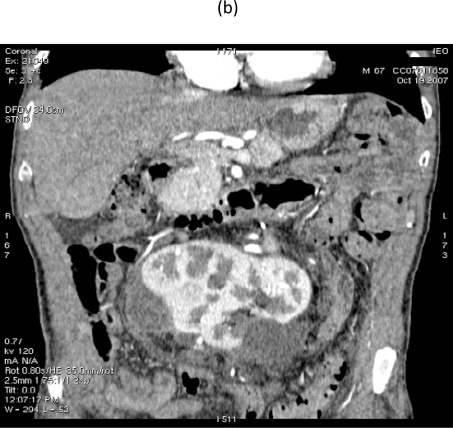
Supernumerary artery for the lower pole of the left kidney was sacrificed during surgery. Follow-up CT images show infarction of the left kidney upper lobe (a); however, the remaining parenchyma of the left kidney was well perfused (b)

On 28 September 2007, at 9.00, a multi-sliced chest and whole abdomen CT scan was performed, both during early and late arterial phases as well as venous phase, for an evaluation of the vessels in view of surgery (Figures 1–5).

**Figure 6:**
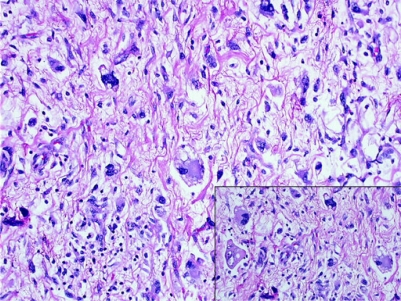
pleomorphic liposarcoma showing highly malignant tumour cells supplied with citoplasmic vacuolization consistent with multivacuolated lipoblasts (inset).

On 28 September 2007, at 14.30, the patient underwent surgery comprising:

1. Cystoscopy with positioning of bilateral ureteral stents.
2. Radical removal of the mass that was separated with difficulty from the horseshoe kidney (the separation was aided by the presence of ureteral stents and by an intra-operative echogram) with binding of one of the three main renal arteries and of the left inferior polar artery (after confirming their transit inside the mass). [ surgical procedure videoclip].
3. During surgery the patient suffered heavy blood loss (22 l of blood) with inadequate haemostasis because of heavy bleeding, requiring splenectomy in an attempt to control the haemorrhage.
4. The patient also suffered hypothermia (34.5 °C), metabolic acidosis, impaired blood clotting, unstable haemodynamics, necessitating suturing with abdominal packing (four packs) for ‘damage control’.
5. The surgical procedure ended at 19.30.
6. The pathology report was high-grade pleomorphic liposarcoma (Figure 6).

The patient was transferred to the ICU for 14 hours, where he underwent:

1. heating with a thermal blanket;
2. correction of metabolic acidosis;
3. transfusion of 37 units of concentrated erythrocytes;
4. transfusion of three pools of platelets, 27 units of fresh plasma and no clotting factors.

On 29 September 2007, at 9.00, the patient (intubated and ventilated) was haemodynamically stable with normal diuresis. After angiography, he underwent second-look surgery with haemostasis and packing removal.

**Figure 7:**
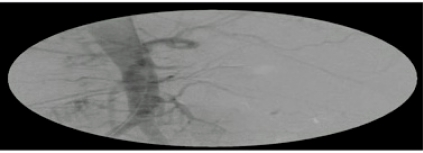
The abdominal aorta is visualized with left renal artery occlusion. The other four renal arteries (one of which arising from common right iliac artery) provide blood supply to the horseshoe kidney, which is ischaemic on the left superior portion http://www.ecancermedicalscience.com/view-article.asp?doi=10.3332/ecancer.2008.77

**Figure 8:**
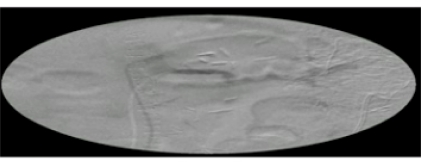
Left renal artery http://www.ecancermedicalscience.com/view-article.asp?doi=10.3332/ecancer.2008.77

**Figure 9:**
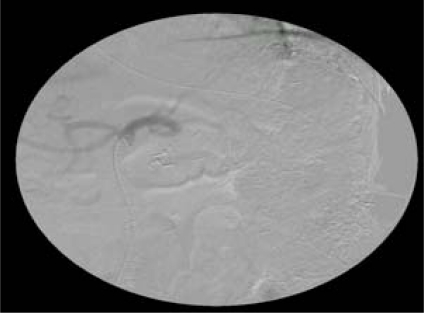
Celiac trunk. Note occlusion of the splenic artery by a clip http://www.ecancermedicalscience.com/view-article.asp?doi=10.3332/ecancer.2008.77

On 29 September 2007, at 9.30, an angiography was performed and no sites of major bleeding were detected (Figures 7–9).

1. Four packs were removed.
2. Haemorrhaging sites were cauterized with argon and infusion of plasma achieving good control of bleeding.
3. The abdominal wall was sutured and two draining tubes were positioned.

Postoperatively, the patient presented:

1. stable haemodynamics without major blood loss from the draining tubes; Hb was stable (9.6 g/dl);
2. normal diuresis.

His ureteral stents were removed after five days, and he required mechanical respiratory support for 10 days due to mild ARDS.

The patient left our ICU after 19 days.

A control CT scan (19 October 2007) demonstrated good vascularization of most of the horseshoe kidney (an infarct area was present to the left upper third) and normal urine flux through the ureters to the bladder.

After discharge (24 October 2007) systemic treatment (Ifosfamide 9 g/sqm + epirubicin 120 mg/sqm + G-CSF) was initiated.

## Comments

1. Retroperitoneal sarcomas require aggressive radical surgery, as there is no alternative adequate treatment.
2. The presence of a horseshoe kidney is a complex surgical problem when a large retroperitoneal mass is present.
3. Damage control with abdominal packing is a viable strategy after removal of a large mass, when bleeding control seems to be difficult after heavy blood loss (22 l in this case), in the presence of hypothermal shock, metabolic acidosis and apparent coagulopathy.
4. Damage control is only possible when an experienced multidisciplinary team (surgeon, anaesthesiologist and internist, all experts in major aggressive surgery and in managing haemorrhagic syndromes occurring in serious polytraumas) and adequate services (e.g. ICU, interventional radiology operational 24 h, transfusion centre and laboratory) are available.

**Definition of damage control:** in the presence of acidosis (pH < 7.3) + hypothermia (<35°C) + coagulopathy with severe haemorrhagic shock (>10 U of blood) [5]:

1. Expeditious surgical control of haemorrhage with temporary abdominal closure.
2. Rewarming and resuscitation in the intensive care unit.
3. Definitive surgical repair of all injuries.
